# Blood–brain barrier leakage at baseline and cognitive decline in cerebral small vessel disease: a 2-year follow-up study

**DOI:** 10.1007/s11357-021-00399-x

**Published:** 2021-06-23

**Authors:** Danielle Kerkhofs, Sau May Wong, Eleana Zhang, Renske Uiterwijk, Erik I. Hoff, Jacobus F. A. Jansen, Julie Staals, Walter H. Backes, Robert J. van Oostenbrugge

**Affiliations:** 1 Department of Neurology, Maastricht University Medical Center+, Oxfordlaan 10, P.O. Box 5800, 6202 AZ Maastricht, The Netherlands; 2 CARIM – School for Cardiovascular Diseases, Maastricht University Medical Center+, Maastricht, The Netherlands; 3 Department of Radiology and Nuclear Medicine, Maastricht University Medical Center+, Maastricht, The Netherlands; 4 MH&Ns – School for Mental Health and Neuroscience, Maastricht University, Maastricht, The Netherlands; 5 Department of Neurology, Zuyderland Medical Centre, Heerlen, The Netherlands

**Keywords:** Cerebral small vessel disease, Blood–brain barrier, MRI, Lacunar stroke, Vascular cognitive impairment, Cognitive decline

## Abstract

Blood–brain barrier (BBB) dysfunction is one of the pathophysiological mechanisms in cerebral small vessel disease (SVD). Previously, it was shown that BBB leakage volume is larger in patients with SVD compared with controls. In this study, we investigated the link between BBB leakage and cognitive decline over 2 years in patients with cSVD. At baseline, 51 patients with clinically overt cSVD (lacunar stroke or mild vascular cognitive impairment) received a dynamic contrast-enhanced MRI scan to quantify BBB permeability in the normal-appearing white matter (NAWM), white matter hyperintensities (WMH), cortical grey matter (CGM), and deep grey matter (DGM). Cognitive function in the domain executive function, information processing speed, and memory was measured in all patients at baseline and after 2 years. The association between baseline BBB leakage and cognitive decline over 2 years was determined with multivariable linear regression analysis, corrected for age, sex, educational level, baseline WMH volume, and baseline brain volume. Regression analyses showed that higher baseline leakage volume and rate in the NAWM and CGM were significantly associated with increased overall cognitive decline. Furthermore, higher baseline leakage volume in the NAWM and CGM, and higher baseline leakage rate in the CGM were significantly associated with increased decline in executive function. This longitudinal study showed that higher BBB leakage at baseline is associated with stronger cognitive decline, specifically in executive function, over 2 years of follow-up in patients with cSVD. These results emphasize the key role of BBB disruption in the pathophysiology and clinical progression of cSVD.

**Supplementary Information:**

The online version contains supplementary material available at 10.1007/s11357-021-00399-x.

## Introduction

Cerebral small vessel disease (cSVD) is an umbrella term that covers several pathologies involving the small vessels in the brain with various etiologies [1]. The most common form is “age- and hypertension-related cerebral microangiopathy” which causes lacunar stroke and contributes importantly to vascular cognitive impairment and vascular dementia [1].

Although the underlying pathophysiological mechanism of cSVD remains largely unclear, dysfunction of the blood–brain barrier (BBB) is thought to be one of the initiating factors [2]. The neurovascular unit (NVU) regulates the close relationship between the BBB and the brain parenchyma [3]. The BBB plays a critical role in maintaining brain homeostasis by regulating the influx of nutrients from the bloodstream, controlling the efflux of wastes and protecting the brain cells from blood constituents by forming a physical barrier [3, 4]. In cSVD, it is thought that dysfunction of the cerebral microvascular endothelium leads to increased permeability of the BBB, causing microstructural brain injury [2]. Recently, it was shown that BBB leakage is more widespread in the brain parenchyma in patients with cSVD compared to healthy controls [5–7]. In addition, cross-sectional studies showed an association between white matter hyperintensity (WMH) volume and increased BBB permeability [8–10]. Postmortem investigations have also revealed an association of BBB permeability with age and WMH load [11, 12].

Whether cognitive deterioration in cSVD is related to BBB dysfunction is unknown. We assume that microstructural brain damage subsequent to increased BBB permeability leads to cognitive decline. However, cross-sectional studies investigating this association are sparse and have yielded inconsistent results [9, 13].

The clinical implications of BBB leakage on cognitive decline in a time-related manner are essential for the understanding of the causal role of BBB leakage in the etiology of cSVD. Longitudinal studies can provide more adequate insights in the temporal relationship between BBB leakage and cognitive decline in cSVD. We aimed to investigate the relationship between baseline BBB leakage and cognitive decline over 2 years in patients with clinically overt cSVD.

## Material and methods

### Study population

We included clinically overt cSVD patients, consisting of patients with lacunar stroke or mild vascular cognitive impairment (mVCI) [5]. Lacunar stroke was defined as an acute stroke syndrome with a compatible recent small subcortical infarct on clinical brain MRI. If no such lesion was visible on MRI or only CT was performed, established clinical criteria for lacunar stroke syndrome were used [14, 15]. Stroke patients were included at least 3 months post-stroke to avoid acute stroke changes. Exclusion criteria included a symptomatic carotid stenosis of ≥ 50% or a possible cardiac embolic source (e.g., atrial fibrillation). Criteria of mVCI were met when patients had (1) subjective complaints of cognitive functioning, (2) objective cognitive impairment in at least one cognitive domain at neuropsychological testing, (3) a Clinical Dementia Rating of ≤ 1 and a Mini-Mental State Examination score of ≥ 20, and (4) vascular lesions on brain MRI that suggested a link between cognitive deficit and cSVD: moderate to severe white matter hyperintensities (WMH; Fazekas score deep > 1 and/or periventricular > 2) or mild WMH (Fazekas score deep = 1 and/or periventricular = 2) combined with lacune(s) or microbleeds [16, 17]. Exclusion criteria included neurodegenerative disease (e.g., Alzheimer’s disease), or in case of severe cognitive impairment defined as a Clinical Dementia Rating of > 1 or a Mini-Mental State Examination score of < 20. General exclusion criteria were other central nervous system diseases or contraindications for MRI.

Patients were included from the Maastricht University Medical Centre and Zuyderland Medical Centre, The Netherlands, between April 2013 and December 2014. Patients underwent brain MRI and neuropsychological assessment at baseline. Neuropsychological assessment was repeated after 2 years. Characteristics of all patients were recorded including age, sex, and the presence of cardiovascular risk factors including hypertension (history of hypertension and/or use of blood pressure–lowering drugs), hypercholesterolemia (history of hypercholesterolemia and/or use of statin), diabetes mellitus (history of diabetes mellitus or use of blood sugar–lowering drugs), smoking (current smoking), and body mass index (BMI: current weight of the subject divided by the square of the current length). Education level was registered and categorized based on the Dutch classification system “Verhage” [18] (in which levels 1, 2, 3, and 4 (primary or low-level secondary school) are considered as low education; level 5 (average-level secondary school) is considered as middle education; and levels 6 and 7 (high level secondary school or university) are considered as high education).

The Medical Ethics Committee of the Maastricht University Medical Center approved the study and informed consent was obtained from all study participants. This study is registered at trialregister.nl (NTR number NTR3786).

### Structural magnetic resonance imaging

All patients underwent structural 3.0 Tesla brain MRI (Achieva TX, Philips Healthcare, Best, The Netherlands). Structural imaging included a T1-weigthed sequence (repetition time/inversion time/echo time TR/TI/TE = 8.3/800/3.8 ms; field of view (FOV) 256 × 256 × 160 mm; 1.0 mm cubic voxel) and a T2-weighted fluid-attenuated inversion recovery (FLAIR) sequence (TR/TI/TE = 4800/1650/299 ms; FOV 256 × 256 × 180 mm; 1.0 mm cubic voxel), which were used for anatomic reference and detection of WMH.

### BBB imaging

Dual-time resolution DCE-MRI was composed by two integrated dynamic sequences differing in dynamic scan time (DST): a fast and a slow sequence [5, 19]. Both sequences were acquired prior to bolus injection. During bolus injection, the fast sequence (DST 3.2 s; TR/TE = 5.6/2.5 ms; FOV: 256 × 200 × 50 mm; voxel size of 2 × 2 × 5 mm; 29 volumes; image acquisition acceleration (SENSE) = 2) was applied to sample the bolus peak and subsequently the slower sequence (DST 30.5 s; TR/TE = 5.6/2.5 ms; FOV: 256 × 256 × 100 mm; voxel size of 1 × 1 × 2 mm; 45 volumes; SENSE = 2) was performed. The contrast agent (gadobutrol; dose 0.1 mmol/kg body weight) was injected in the antecubital vein at a rate of 3 ml/s using a power injector. To convert the contrast-enhanced signal intensities to concentrations in tissue, T1 mapping was performed before contrast agent infusion and DCE-MRI [20].

### Image processing

FreeSurfer [21] software was used for the segmentation of grey and white matter from the T1-weighted images and subsequently WMH were delineated from the normal-appearing white matter (NAWM) on the FLAIR images using a semi-automated segmentation tool, with visual inspection and manual correction [22]. FLAIR and T1-weighted images were spatially co-registered (FSL [23], v5.0) [23] and the following brain regions were selected: NAWM, WMH, cortical grey matter (CGM), and deep grey matter (DGM). The volume of the brain and the WMH was normalized to the intracranial volume to calculate relative brain volume and the relative WMH volume.

### Pharmacokinetic modelling and histogram analysis

For the analysis of the DCE-MRI data, we performed pharmacokinetic modelling and histogram analysis to calculate BBB leakage measures, as described before [5]. The concentration of the contrast agent was calculated by using the relative signal enhancement and T1 mapping [20]. Blood signal in the superior sagittal sinus was used for the vascular input function to calculate the concentration in blood plasma [24]. Subsequently, the graphical Patlak graphical method [25] was used to calculate the leakage rate in terms of the transfer constant *K*_i_ (min^− 1^). *K*_*i*_ was determined in a voxel-wise manner and a histogram was obtained for the *K*_i_ values in each brain region. To correct for noise, we mirrored the negative *K*_i_ values to the positive axis and subtracted both parts from the original *K*_i_ distribution, resulting in a positive histogram with *K*_i_ values reflecting the detectable leakage rates. Quantitative BBB leakage measures were obtained using this histogram: mean *K*_i_, indicating the leakage rate was calculated as the mean of all the positive noise-corrected voxels, and leakage volume (*v*_L_) as the remaining area under the histogram curve, representing the fractional volume of leaky brain tissue that can be detected (i.e., the spatial extent of leakage) [26].

### Neuropsychological assessment

Cognitive performance covering three main cognitive domains was assessed at baseline and after 2 years of follow-up, as described before [27]. Memory domain was measured with Rey Auditory Verbal Learning Test (immediate recall, delayed recall, and delayed recognition) and the Digit Span Forward (subtest of Wechsler Adult Intelligence Scale (WAIS)-III) [28, 29]. Executive function domain was tested using the Stroop Color-Word Test interference score (SCWT) (time of part B minus time of part A), Trail Making Test (TMT) interference score (time of part B minus time of part A), Category (animals and professions) and Letter Fluency, Letter-Number Sequencing (subtest of WAIS-III), and Digit Span Backward (subtest of WAIS-III) [29–33]. Information processing speed was determined using the Symbol Substitution-Coding (subtest of WAIS-III), TMT part A, and SCWT parts 1 and 2 [29–31]. Alternate versions were used for baseline and follow-up assessment for the Rey Auditory Verbal Learning Test and the Letter Fluency test.

For each patient, raw test scores at follow-up were subtracted from test scores at baseline. The scores of tests with higher scores representing worse performance (i.e., SCWT and TMT) were inverted. These raw decline scores were transformed into standardized values (z-scores), by dividing the difference between the individual raw decline score and the overall group sample mean by the overall group sample standard deviation (SD). Cognitive decline compound scores were determined by averaging the z-scores of all tests within one domain, and higher compound scores indicated stronger decline. In addition, an overall cognitive decline compound score was calculated by averaging the three domain compound scores. When one test score was missing, compound scores were calculated from the scores of the remaining tasks. In four patients, more than one test score in the executive domain was missing. For these patients no reliable domain score could be calculated and consequently also the overall cognition decline score was missing.

### Statistical analysis

To examine the associations between BBB leakage at baseline and cognitive decline, we used univariable linear regression analyses, with the compound scores of the different cognitive domains as dependent variable, and leakage volume and rate as independent variable. Next, multivariable linear regression analysis was performed with adjustments for age, sex, educational level, baseline relative WMH volume, and baseline brain atrophy (relative brain volume). Statistical analysis was performed using SPSS 22.0. Results were considered significant at *P* < 0.05.

## Results

At baseline, we included 80 patients with cSVD who underwent brain MRI and neuropsychological assessment. Baseline images were not suitable for analysis in 2 patients, and during the follow-up period, 4 patients died and 23 patients were not willing to participate in the follow-up study. This gave 51 patients who completed the follow-up neuropsychological assessment after a mean of 2.12 years (SD ± 0.11) and were included in the present analysis.

The characteristics of the included patients and lost to follow-up patients are presented in Table 1. The follow-up group included more lacunar stroke patients (71% versus 28%, respectively, *P* < 0.001) and patients who completed follow-up were younger (66.7 ± 11.9. versus 75.1 ± 6.9 years, respectively, *P* = 0.001), had less often diabetes (8% versus 28%, respectively, *P* = 0.017), and less often coronary artery disease (12% versus 31%, respectively, *P* = 0.034). Leakage volume was higher in some brain regions of the patients lost to follow-up. Furthermore, the included patients had better overall cognition scores at baseline (*P* = 0.013) compared to patients who did not complete follow-up. For cognitive scores on all individual test at baseline and follow-up, please see the supplemental Table I. Baseline leakage measures for lacunar stroke patients and VCI patients are presented in supplemental Table II.

**Table 1:** Characteristics at baseline of the included patients and lost to follow-up patients

Demographics and risk factors	Included in follow-up n** = **51	Lost to follow-up n** = **29	P-value
Age, years (SD)	67 (12)	75.1 (7)	0.001*
Male (%)	30 (59)	16 (55)	0.755
Lacunar stroke/mVCI (%)	36 (71)/ 15 (29)	8 (28)/21 (73)	< 0.001*
Educational level (%)			0.385
Low	24 (47)	11 (38)	
Average	18 (35)	11 (38)	
High	9 (18)	7 (24)	
Hypertension (%)	33 (65)	18 (62)	0.816
Hypercholesterolemia (%)	29 (60)	16 (55)	0.885
Diabetes (%)	4 (8)	8 (28)	0.017*
BMI kg/m^2^ (SD)	26 (4)	25 (4)	0.493
Coronary artery disease (%)	6 (12)	9 (31)	0.034*
Peripheral artery disease (%)	2 (4)	3 (10)	0.259
Smoking (%)	14 (28)	5 (17)	0.308
Time to follow-up, months (SD)	26 (1)	-	
MRI measures			
Relative brain volume (SE)	0.675 (0.007)	0.654 (0.008)	0.633
Relative WMH volume (SE)	0.014 (0.002)	0.016 (0.003)	0.058
Total WMH Fazekas score, median (IQR)	4 (2–6)	4 (3–6)	0.197
Silent lacunar infarct (%)	26 (51)	12 (41)	0.588
Microbleeds (%)	12 (24)	6 (21)	0.898
BBB leakage volume: v_*L*_ (%)			
NAWM (SD)	32 (16)	41 (16)	0.030*
WMH (SD)	39 (19)	48 (20)	0.067
CGM (SD)	18 (11)	24 (13)	0.035*
DGM (SD)	30 (19)	40 (17)	0.030*
BBB leakage rate: *K*_i_ (10^−4^ min^−1^)			
NAWM (SD)	3.1 (1.6)	3.6 (1.4)	0.195
WMH (SD)	3.4 (2.0)	3.9 (1.9)	0.280
CGM (SD)	2.3 (1.2)	2.4 (1.1)	0.069
DGM (SD)	3.1 (2.0)	3.7 (2.0)	0.155

*SD*, standard deviation; *SE*, standard error; *IQR*, interquartile range; *BMI*, body mass index; *NAWM*, normal-appearing white matter; *WMH*, white matter hyperintensities; *CGM*, cortical grey matter; *DGM*, deep cortical grey matter; *Brain volume*, brain volume normalized to intracranial volume; *WMH volume*, WMH volume normalized to intracranial volume. Total Fazekas score: Periventricular (0–3) and deep (0–3) WMH scores were summed. * P < 0.05

### *BBB leakage volume (v*_*L*_*) and cognitive decline*

In Table 2, we present the adjusted associations between baseline leakage volume *v*_L_ and cognitive decline (unadjusted results can be found in supplemental Table III). After correction for age, sex, educational level, baseline relative WMH volume, and baseline relative brain volume, a higher *v*_L_ in both NAWM and CGM was associated with increased decline scores on overall cognition (respectively B = 0.72, *P* = 0.023; B = 1.40, *P* = 0.001) and executive function (respectively B = 1.29, *P* = 0.012; B = 2.11, *P* = 0.003). In the WMH, a higher baseline leakage volume was associated with stronger decline in the executive domain (B = 0.92, *P* = 0.031).

**Table 2:** Associations between baseline BBB leakage volume and rate and cognitive decline

	Overall		Executive		Speed		Memory	
	Adjusted* B (95% CI)	*P*-value	Adjusted* B (95% CI)	*P*-value	Adjusted* B (95% CI)	*P*-value	Adjusted* B (95% CI)	*P*-value
*v*_L_								
NAWM	0.72 (0.10–1.34)	0.023^†^	1.29 (0.30–2.28)	0.012^†^	− 0.29 (− 1.59–1.01)	0.652	− 0.01 (− 1.32–1.29)	0.982
WMH	0.43 (− 0.09–0.96)	0.102	0.92 (0.09–1.75)	0.031^†^	− 0.49 (− 1.55–0.57)	0.353	− 0.15 (− 1.22–0.92)	0.782
CGM	1.40 (0.59–2.21)	0.001^†^	2.11 (0.77–3.46)	0.003^†^	0.56 (− 1.36–2.49)	0.590	1.49 (− 0.25–3.24)	0.092
DGM	0.28 (− 0.26–0.82)	0.298	0.83 (− 0.02–1.68)	0.056	− 0.51 (− 1.60–0.57)	0.344	− 0.23 (− 1.33–0.86)	0.668
*K*_i_								
NAWM	0.65 (0.065–1.23)	0.030^†^	0.80 (− 0.18–1.78)	0.106	− 0.25 (− 1.51–1.01)	0.687	0.22 (− 1.04–1.48)	0.730
WMH	0.43 (− 0.05–0.92)	0.078	0.59 (− 0.21–1.39)	0.145	− 0.49 (− 1.51–0.53)	0.339	0.15 (− 0.88–1.18)	0.768
CGM	1.34 (0.64–2.00)	< 0.001^†^	1.90 (0.71–3.11)	0.003^†^	0.61 (− 1.13–2.35)	0.484	1.86 (0.33–3.34)	0.018^†^
DGM	0.31 (− 0.20–0.81)	0.223	0.62 (− 0.19–1.42)	0.132	− 0.52 (− 1.54–0.49)	0.306	− 0.14 (− 1.17–0.89)	0.784

B values represent standardized regression coefficients. v_*L*_, leakage volume (%); *K*_*i*_, leakage rate (10^−3^ min^−1^); *CI*, confidence interval; *NAWM*, normal-appearing white matter; *WMH*, white matter hyperintensities; *CGM*, cortical grey matter; *DGM*, deep cortical grey matter

^*^Adjusted for age, sex, educational level, baseline relative WMH volume and baseline brain volume

^†^*P* < 0.05

### *BBB leakage rate (K*_*i*_*) and cognitive decline*

The adjusted associations between baseline leakage rate K_i_ and cognitive decline are shown in Table 2 (unadjusted results can be found in supplemental Table IV). After correction for age, sex, education level, baseline relative WMH volume, and baseline relative brain volume, an increased leakage rate at baseline in the CGM was significantly associated with increased decline scores on overall cognition (B = 1.34, *P* < 0.001), executive function (B = 1.90, *P* = 0.003), and memory (B = 1.86, *P* = 0.018). Furthermore, a higher baseline leakage rate in NAWM was associated with increased decline for overall cognition scores (B = 0.65, *P* = 0.030).

## Discussion

In patients with clinically overt cSVD, we found that both higher baseline BBB leakage volume and leakage rate in white and grey matter structures are associated with stronger overall cognitive decline. Particularly, decline in executive function was associated with increased baseline leakage volume and rate in the CGM.

The BBB is thought to play a pivotal role in the pathogenesis of cSVD and disease progression [5, 7, 8, 10]. In a previous study with DCE-MRI, we were able to measure very subtle BBB leakage rates and we introduced the concept of leakage volume measurement [5, 19]. We found an increased volume of subtle leakage in the CGM, NAWM, and WMH of cSVD patients compared to controls [5]. However, we, and others, found no cross-sectional association between BBB leakage and cognitive impairment in patients with cSVD [9, 10]. The differences in the results between the cross-sectional and the present longitudinal study indicate that BBB leakage and cognitive function are associated in a time-related manner. The association between macrostructural MRI markers of cSVD, such as WMH, and cognitive decline is well-known [34, 35]. However, we focused on the presumed early microvascular damage reflected by BBB leakage instead of late-stage morphological MRI markers. It is assumed that subtle BBB leakage plays an early role in the pathophysiology and precedes loss of brain tissue integrity which accumulates and then leads to worsening of cognitive function. The observed time-related association of baseline BBB leakage parameters in different brain regions is in line with the upcoming view that cSVD is a disease with diffuse microvascular damage not restricted to white and deep grey matter [15].

We found the most robust associations between BBB leakage and cognitive decline in the cortical grey matter. Although cSVD classically is considered a subcortical disease, this and other studies show that the cortex is involved in the pathophysiology of cSVD function [36–39]. Previous studies reported that cortical microinfarcts, cortical thinning, and reduction of capillaries are involved in cSVD and are related to decreased cognitive function [36–39]. We now showed that also change in BBB permeability in the cortex was associated with cognitive decline in cSVD patient.

In addition, we found that leakage of the BBB in the NAWM was associated with an overall decline in cognitive performance. This might indicate that impairment of the BBB in normal-appearing brain tissue is involved in the progression of cSVD even before morphological abnormalities occur in this NAWM. Within WMH, the tissue architecture and vasculature may already be structurally so disturbed that there is a ceiling effect of BBB leakage and no association can be shown with cognitive decline. Indeed, leakage volume and leakage rate were highest within WMH. The other single significant test results (association between leakage volume in WMH and executive function, and between leakage rate in CGM and memory) also fit in this picture, though it cannot be excluded that they resulted from chance (e.g., due to the limited sensitivity of the BBB imaging and/or the multiple testing) and we need to be more cautious on more detailed conclusions.

Our results showed particularly an association between BBB leakage and decline in executive function, but not in processing speed and working memory. From our results, it appeared that the scores on executive function, in contrast to processing speed or memory, declined most strongly. In a previous study, it has been shown that an composite measure of executive function is the most sensitive measure to test cognitive decline in cSVD patients [40]. Furthermore, previous studies investigating the association between imaging markers and cognitive decline in cSVD patients also found the strongest association for executive function [40, 41]. Impairment in executive function seems to be a prominent and early feature of vascular cognitive decline in the investigated age range [42].

To our knowledge, so far, only one other study investigated a longitudinal association between BBB leakage and cognitive decline in cSVD patients [10]. In that DCE-MRI study in patients with mild ischemic lacunar or cortical stroke, it was found that only leakage in the WMH predicted declining cognition at 1 year follow-up, using a brief cognitive screening test [10]. Differences in the composition of the study population, the use of a more extensive cognitive test battery, and different DCE technique and/or the longer follow-up period (2 years) in our study could explain the fact that we found significant associations between the BBB leakage in NAWM and CGM but not WMH, and cognitive decline. In patients with Alzheimer’s disease, it was suggested that accelerated breakdown of the BBB by apolipoprotein E4 is associated with cognitive decline independently of the Alzheimer’s pathology [43]. Furthermore, also in a healthy patient sample, it was shown that BBB leakage in both white and grey matter is related to decline in memory function over a long follow-up period of 12 years [44]. Our results are in line with these previous studies and further emphasize the key role of the BBB in cognitive deterioration, independent of effects due to age.

The major strengths of our study were the strict clinical-radiological definition of cSVD, the longitudinal design with a 2-year follow-up period, and the use of an extensive neuropsychological assessment, enabling us to examine three different cognitive domains. Furthermore, we have examined both leakage volume and leakage rate as measures for BBB leakage. Leakage volume is a relative new measure; we previously found that leakage volume is higher in cSVD patients compared to healthy controls [5], suggesting that BBB leakage volume is a sensitive measure for BBB leakage.

This study also has limitations. A relatively large number of more severely diseased patients were lost to follow-up which limits the generalizability of the study. However, the small sample size, biased to younger, and cognitively better patients with less cardiovascular risk factors most likely has resulted in an underestimation of the found associations. Furthermore, the follow-up period for cognitive assessment was relatively short which may have led to relative low decline in cognition and therefore underestimation of the associations.

The second limitation is the heterogeneous clinical features of the cSVD patients in the study. We included both lacunar stroke patients and VCI patients. However, the underlying small vessel pathology is presumed to be the same in these groups. Furthermore, we used clear in- and exclusion criteria to prevent as much as possible that there were other causes for the lacunar stroke (e.g., atrial fibrillation, carotid stenosis) or cognitive impairment (e.g., Alzheimer’s disease). Also, none of the patients had a clinical stroke during the follow-up period. The study population was mainly Caucasian. We therefore do not know if the results can be generalized to other races. Furthermore, we only investigated cSVD patients. For future studies, it would be of interest to investigate the association between BBB permeability and cognitive decline in healthy controls. Another potential limitation of this study is that we only assessed BBB permeability at baseline. Therefore, we have no indication of the change in BBB permeability over 2 years follow-up and the potential contribution of any changes in these 2 years to cognitive decline and change in brain volume, which would be of interest for future studies.

In conclusion, this longitudinal study showed that higher baseline BBB leakage is associated with stronger cognitive decline over 2 years of follow-up in patients with clinical overt cSVD. Our results provide more insight in the early role of BBB leakage in the pathophysiology and progression of cSVD. Future exploration of the complex relationship between BBB leakage and the progression of cSVD including cognitive decline is indicated.

## Supplementary Information

Below is the link to the electronic supplementary material.

Supplementary file1 (DOCX 24.6 KB)

## Data Availability

The data that support the findings of this study are available from the corresponding author upon reasonable request.

## Abbreviations

BBB: Blood–brain barrier
CGM: Cortical grey matter
cSVD: Cerebral small vessel disease
DCE: Dynamic contrast-enhanced
DGM: Deep grey matter
DST: Different dynamic scan time
FLAIR: Fluid-attenuated inversion recovery
FOV: Field of view
mVCI: Mild vascular cognitive impairment
NAWM: Normal-appearing white matter
NVU: Neurovascular unit
TE: Echo time
TI: Inversion time
TR: Repetition time
WMH: White matter hyperintensities
