# Supplementary material for: Blood–brain barrier leakage at baseline and cognitive decline in cerebral small vessel disease: a 2-year follow-up study
Source: GeroScience. 2021 Jun 23;43(4):1643–52. doi: 10.1007/s11357-021-00399-x (PMC8492799; doi:10.1007/s11357-021-00399-x)
Supplement: Supplementary file 1 — Supplementary file1 (DOCX 24.6 KB) [file 11357_2021_399_MOESM1_ESM.docx]

**Blood-brain barrier leakage and cognitive decline in cerebral small vessel disease: a 2-year follow-up study**

Danielle Kerkhofs^1,2^, MD; Sau May Wong^3,4^, PhD; Eleana Zhang^1,2,4^, MD, PhD; Renske Uiterwijk^1,4^, PhD; Erik I. Hoff^5^, MD, PhD; Jacobus F.A. Jansen^3,4^, PhD; Julie Staals^1,2^, MD, PhD; Walter H. Backes^2,3,4^, PhD; Robert J. van Oostenbrugge^1,2,4^, MD, PhD

Supplemental Table I. Neuropsychological test scores at baseline and follow-up

Supplemental Table II. Leakage measures for lacunar stroke and vascular cognitive impairment patients

Supplemental Table III. Association between baseline BBB leakage volume and cognitive decline

Supplemental Table IV. Association between baseline BBB leakage rate and cognitive decline

**Online Supplement**

Supplemental Table I. Neuropsychological test scores at baseline and follow-up

|  | **Baseline**  **Mean Score (SD)** | **Follow-up**  **Mean Score (SD)** |
| --- | --- | --- |
| Stroop Colour-Word Test Part 1# | 57.9 (16.3) | 62.9 (20.5) |
| Stroop Colour-Word Test Part 2# | 74.7 (20.0) | 83.5 (28.1) |
| Stroop Colour-Word Test Part 3# | 152.0 (66.4) | 156.1 (67.1) |
| Rey Auditory Verbal Learning Test |  |  |
| Immediate recall | 35.1 (10.1) | 36.4 (11.6) |
| Delayed recall | 6.3 (3.5) | 6.7 (3.4) |
| Delayed recognition | 12.2 (2.3) | 11.6 (3.9) |
| Trail Making Test A# | 58.9 (27.8) | 63.1 (36.8) |
| Trail Making Test B# | 143.4 (87.4) | 159.2 (104.0) |
| Category Fluency | 32.3 (10.6) | 31.3 (11.7) |
| Letter Fluency | 26.1 (12.6) | 25.1 (11.8) |
| Symbol Substitution - Coding | 46.7 (18.4) | 43.9 (21.1) |
| Digit Span Forward | 7.7 (2.1) | 7.4 (1.8) |
| Digit Span Backward | 5.3 (1.7) | 5.1 (1.7) |
| Letter Number Sequencing | 7.8 (3.0) | 7.6 (3.1) |

# Test score units is in seconds

Supplemental Table II. Leakage measures for lacunar stroke and vascular cognitive impairment patients

|  | **Lacunar stroke (n = 36)** | **VCI (n = 15)** |
| --- | --- | --- |
| **BBB leakage volume : v*_L_* (%)** |  |  |
| NAWM (SD) | 34 (16) | 31 (16) |
| WMH (SD) | 38 (19) | 42 (20) |
| CGM (SD) | 17 (11) | 20 (10) |
| DGM (SD) | 28 (18) | 36 (20) |
| ***BBB leakage rate: K*_i_ (10^-4^min^-1^)** |  |  |
| NAWM (SD) | 3.2 (1.6) | 2.9 (1.7) |
| WMH (SD) | 3.5 (2.0) | 3.3 (2.0) |
| CGM (SD) | 2.1 (1.2) | 2.6 (1.1) |
| DGM (SD) | 2.9 (2.0) | 3.4 (2.0) |

v_L,_ Leakage volme; *K*_i_, Leakage rate; NAWM, Normal appearing white matter; WMH, White matter hyperintensities; CGM, Cortical grey matter; DGM, Deep cortical grey matter; VCI, vascular cognitive impairment

Supplemental Table III. Association between baseline BBB leakage volume and cognitive decline

|  |  | **Overall** | | **Executive** | | **Speed** | | **Memory** | |
| --- | --- | --- | --- | --- | --- | --- | --- | --- | --- |
| v_L_ | Model | B (95% CI) | *P*-value | B (95% CI) | *P*-value | B (95% CI) | *P*-value | B (95% CI) | *P*-value |
| NAWM | Unadjusted | 0.28  (-0.50 – 1.01) | 0.480 | 0.78 (-0.22 – 1.79) | 0.123 | -0.83 (-2.19 – 0.53) | 0.225 | -0.40 (-1.65 – 0.86) | 0.529 |
|  | Adjusted* | 0.72  (0.10 – 1.34) | 0.023† | 1.29  (0.30 – 2.28) | 0.012† | -0.29  (-1.59 – 1.01) | 0.652 | -0.01  (-1.32 – 1.29) | 0.982 |
| WMH | Unadjusted | 0.26  (-0.36 – 0.88) | 0.402 | 0.75  (-0.05 – 1.54) | 0.064 | -0.48 (-1.59 – 0.64) | 0.394 | -0.24  (-1.26 – 0.78) | 0.636 |
|  | Adjusted* | 0.43  (-0.09 – 0.96) | 0.102 | 0.92  (0.09 – 1.75) | 0.031† | -0.49  (-1.55 – 0.57) | 0.353 | -0.15  (-1.22 – 0.92) | 0.782 |
| CGM | Unadjusted | 0.93  (-0.09 – 1.94) | 0.074 | 1.65  (0.35 – 2.94) | 0.014† | 0.19  (-1.75 – 2.13) | 0.842 | 0.80  (-0.89 – 2.49) | 0.347 |
|  | Adjusted* | 1.40  (0.59 – 2.21) | 0.001† | 2.11  (0.77 – 3.46) | 0.003† | 0.56  (-1.36 – 2.49) | 0.590 | 1.49  (-0.25 – 3.24) | 0.092 |
| DGM | Unadjusted | 0.09 (-0.56 – 0.73) | 0.792 | 0.57  (-0.26 – 1.41) | 0.173 | -0.49 (-1.65 – 0.67) | 0.401 | -0.46 (-1.52 – 0.60) | 0.388 |
|  | Adjusted* | 0.28  (-0.26 – 0.82) | 0.298 | 0.83  (-0.02 – 1.68) | 0.056 | -0.51  (-1.60 – 0.57) | 0.344 | -0.23  (-1.33 – 0.86) | 0.668 |

B values represent standardized regression coefficients. v_L,_ Leakage volme (%); CI, confidence interval; NAWM, Normal appearing white matter; WMH, White matter hyperintensities; CGM, Cortical grey matter; DGM, Deep cortical grey matter.
*Adjusted for age, sex, educational level, relative WMH volume and brain volume.
**†** *P*<0.05

Supplemental Table IV. Association between baseline BBB leakage rate and cognitive decline

|  |  | **Overall** | | **Executive** | | **Speed** | | **Memory** | |
| --- | --- | --- | --- | --- | --- | --- | --- | --- | --- |
| *K*_i_ | Model | B (95% CI) | *P*-value | B (95% CI) | *P*-value | B (95% CI) | *P*-value | B (95% CI) | *P*-value |
| NAWM | Unadjusted | 0.34 (-0.39 – 1.07) | 0.358 | 0.51  (-0.45 – 1.47) | 0.288 | -0.80  (-0.21 – 0.52) | 0.229 | -0.10  (-1.32 – 1.11) | 0.865 |
|  | Adjusted* | 0.65  (0.065-1.23) | 0.030† | 0.80  (-0.18 – 1.78) | 0.106 | -0.25  (-1.51 – 1.01) | 0.687 | 0.22  (-1.04 – 1.48) | 0.730 |
| WMH | Unadjusted | 0.24  (-0.37 – 0.86) | 0.432 | 0.43 (-0.37 – 1.24) | 0.285 | -0.77 (-1.87 – 0.33) | 0.163 | -0.05  (-1.07 – 0.97) | 0.919 |
|  | Adjusted* | 0.43  (-0.05 – 0.92) | 0.078 | 0.59  (-0.21 – 1.39) | 0.145 | -0.49  (-1.51 – 0.53) | 0.339 | 0.15  (-0.88 – 1.18) | 0.768 |
| CGM | Unadjusted | 1.05  (0.11 – 1.98) | 0.029 | 1.69 (0.50 – 2.88) | 0.007† | 0.31 (-1.51 – 2.14) | 0.731 | 1.30  (-0.27 – 2.86) | 0.102 |
|  | Adjusted* | 1.34  (0.64 – 2.00) | <0.001† | 1.90 (0.71 – 3.11) | 0.003† | 0.61  (-1.13 – 2.35) | 0.484 | 1.86  (0.33 – 3.34) | 0.018† |
| DGM | Unadjusted | 0.16 (-0.45 – 0.76) | 0.605 | 0.41  (-0.38 – 1.20) | 0.299 | -0.57  (-1.66 – 0.51) | 0.294 | -0.33 (-1.33 – 0.66) | 0.505 |
|  | Adjusted* | 0.31  (-0.20 – 0.81) | 0.223 | 0.62  (-0.19 – 1.42) | 0.132 | -0.52  (-1.54 – 0.49) | 0.306 | -0.14  (-1.17 – 0.89) | 0.784 |

B values represent standardized regression coefficients. *K*_i_, Leakage rate (10^-3^min^-1^); CI, confidence interval; NAWM, Normal appearing white matter; WMH, White matter hyperintensities; CGM, Cortical grey matter; DGM, Deep cortical grey matter.
*Adjusted for age, sex, educational level, relative WMH volume and brain volume.
**†** *P*<0.05
